# Principles and Practice of Surgery

**Published:** 1848-11

**Authors:** 


ART. III.—*Principles and Practice of Surgery.* By the late George M'Clellan, M. D. Edited by his son, John H. B. M'Clellan, M. D. Philadelphia; Grigg, Elliot & Co., No. 14, North Fourth St. 1848. (*Continued from page* 214.)

In the general chapter entitled "Effects of Injuries upon the Blood Vessels," no new theories are advanced, but the reader is left to infer that the author adopts completely the old doctrines of Hunter in relation to the pathology of inflammation, and that he regards inflammation as an "increased" action, and some of its forms as "healthy" actions.

"Redness, heat, swelling and pain," are the usual signs of inflammation, and are explained by Dr. M'Clellan in the usual manner, except that he sees no good reason why the change of the capillaries from colorless to red, should be ascribed to the admission of larger and red globules, since the whole circumstance is completely explicable by the fact that "the blackest fluid will look pale and even transparent when contained in very fine glass tubes." The change to red is owing to the larger amount of blood which the capillaries are now conveying, and not to the different size, or color of the globules.

Our author still continues to exhibit his partiality for the doctrines of Hunter, when speaking of the changes in the blood produced by inflammation, and contends for the heresy of *vitality,* with a confidence and boldness, which are, at this day, almost startling. He thus concludes:

"We may say, in support of the great Hunterian doctrine, therefore, that the presence of coagulating lymph in every drop of the circulating fluid confers upon the blood the powers of life, even if we do not agree with the latest microscopists in regarding the two orders of globules as organized substances."

In the treatment of the "inflammatory fever" sometimes from injuries, &c., M'Clellan declares for prompt and active blood-letting.

"Notwithstanding all we have said respecting the danger of indiscriminate blood-letting in cases of mere plethoric or tumultuous excitement, there can be no doubt of its safety or efficacy in decisive inflammations. The system undergoes a remarkable change under the influence of inflammation, in consequence of which blood-letting can be borne even by persons of the feeblest constitution."

"In severe inflammations, however, and especially of the internal or vital organs, repeated and large bleedings are frequently required. The first operation will, in these cases, sometimes raise the pulse instead of lowering it, and develope a great increase of the excitement. The oppression from the overloaded venous sinuses and auricles of the heart being removed by the first blood-letting, the action of the heart becomes more free and vigorous, and then an apparent increase of the disease follows.

This fact was not understood by the celebrated Louis when he asserted that blood-letting, during the first four days of a peripneumony, always proved injurious. He bled but once each day, and did not afterwards resort to the proper means of overcoming the subsequent reactions. In all such cases the patient should be watched incessantly; and the orifice be reopened from hour to hour, and a repetition of the blood-letting enforced as often as the pulse rises, until finally all efforts at reaction are overcome. In this way alone the most violent and dangerous inflammations of the internal organs can be subdued. Of course all the adjuvant or accessory means of treatment should at the same time be actively employed. The severe cases of peripneumonia, in this country, would all die or pass beyond redemption without such treatment."

Mercury is regarded as one of the most important adjuvants to bleeding, but it is a Samson to do evil, as well as to do good, and its pernicious effects are especially seen when used as a constitutional remedy in the earlier stages of acute diseases. Antimony, also, in minute and continued doses, is one of our most direct sedatives, and is not generally to be omitted as an antiphlogistic. Opium and morphine have also their appropriate places in certain inflammations.

Among the topical remedies no single one occupies so important a position as the nitrate of silver, which was used by the Dr. not only in all the various forms of inflammations in which practitioners have generally employed it, but also as an application over joints suffering under a deep seated inflammation, over the side in pleuritis, and in short, upon the skin, wherever, the subjacent tissues were inflamed.

Suppuration, ulceration and granulation, follow inflammation. Pus is regarded as an actual secretion, and incapable of softening other structures, but it is noticable that in certain situations, as about the anus, &c., cavities containing pus are often highly offensive when first opened; a circumstance which must be ascribed to the power of endosmosis possessed by the textures surrounding the cavity. Such abscesses cannot be opened too early.

*Granulation* becomes another text upon which the splendid investigations of Hunter are made to compare with the supposed novelties of modern speculators, and the "great investigator of life," is shown to have anticipated almost all that can be considered as probable in the new "cell doctrine." The refined extension of this new theory is thus severely rebuked, and it now needs only the satire of a Swift, or a Butler, to insure for it immortality.

"We must, then, according to the doctrine, have passed during the first six months of uterine existence through all the forms and assumed all the types of the whole of our Pre-Adamic ancestry, from the earliest monad up to the first man himself.

But enough of this folly. The cell-doctrine has already been carried too far for the sober sense of practical surgeons. Let it remain as a plaything for the fancy builders in science. Allow the microscopists to pursue their own researches, and work out their favorite results. "The tools to him who can handle them." We will follow the investigation of plain and tangible things, which we can see with our own eyes, and manage with the hands which God has given to us. It will add no strength to the certainty of our knowledge to decide whether a small particle of lymph be in the shape of a globule or a cyst before it becomes the medium of organization. The archetypes of Plato were just as important to the Baconian philosophy as such matters are to the progress of modern surgery. The chemists have found out that it is not necessary to ascertain the mathematical shapes of the integral molecules of matter, in order to study their laws of arrangement and combination. So we can go on with our pathological inquiries on the broad foundation laid down by Hunter, without stopping to question whether the three primitive monads were cubes, or tetrahedrons, or spheres."

The sections treating of cicatrization, hectic fever, and irritative fever, mortification, and erysipelas in its various forms, will amply repay for the reading, but we have been especially interested in the graphic description given of gangrenous erysipelas.

"During the harvest season of our northern latitudes, sporadic cases of this terrible malady sometimes occur among intemperate laborers who work from sunrise to sunset in the open air, and under an almost tropical sun. Stimulated by high daily wages, and the most pernicious kinds of alcoholic liquors, such men sometimes overtask their powers, and inflame their systems to the highest pitch of exasperation. The food which they consume at this season of the year is, moreover, especially unwholesome. It consists almost exclusively of badly cured salt meats, and heavy Indian corn bread, with the half decayed vegetables of the preceding year, combined with a harsh drink of fermented apple-juice, called cider, pickles, and a plenty of fresh milk.* It naturally follows, that intemperance combined with such diet and fatiguing exposure to an ardent sun for fourteen

* "When I spoke of this subject in a surgical lecture at one of the medical colleges in this city, several years ago, it happened that a very intelligent old gentleman from the interior of New England, was in attendance in company with his son, then a student of medicine. The old gentleman stopped to converse with me after the lecture, and stated that he had personally witnessed three such cases of sudden mortification, and heard of four others in his region of the country within a period of twenty years. They all occurred in harvest and mowing time, in the irregular sort of laborers, who frolic about the country at most seasons, and let themsefves out by the day at high wages in harvest time. He stated the difficulty of keeping any kind of fresh provisions over a very few hours in the hot summer days and nights, and want of all new vegetables in that time of the year, to be the great cause of the use of such unwholesome diet as I have described. In the northern states, no summer vegetable produce ripens so early as harvest time, and the laborers are compelled to feed on half decayed or sprouted potatoes, beets, cabbages, &c., of the preceding year. Of late, however, I understand that more attention has been paid in the north, to the cultivation of early spring vegetables and fruits, and that the use of wheat flour has become more common there among the laboring people. But in all probability, the great and glorious temperance movement of the day has done most towards checking the development of such cases as I have described."

hours every day in succession, must tend to the production of the most dangerous state of constitutional irritability. Then the slightest prick or contusion will always be followed by a widely diffused inflammation. But occasionally, an overwhelming and speedily fatal explosion of constitutional and local disturbance will supervene. A mower who has a short time before struck his foot against the sharp point of a twig, or a reaper who has merely scratched a finger with a stub of straw, will throw down his implement of labor. and scream with mortal agony. The injured member will suddenly tumefy, and present a glazy appearance with livid streaks, intermingled with green and yellow patches. Gangrenous vesicles will soon form, and putrescent odor begin to exhale. The pulse will be feeble and tremulous, the skin cold, and the respiration hurried and moaning. The face will be contracted, or hippocratic; the breath putrid, the eyes glairy, and the epigastric region distressed with a sense of horrible oppression. In from twelve to twenty-four hours the poor wretch will die, with the limb enormously swollen, tense, and crackling, with all the appearances of a malignant erysipelas, terminating in gangrene. No treatment has ever been known to check the progress of this dissase. The tumefaction and lividity will, in the meantime, have extended up to the very trunk of the body so as apparently to have invaded the vitals. The laborious fishermen on our coast, especially those who are addicted to intemperance, and still more coarse and indigestible fare than the agricultural laborer of the same class, are said to be occasionably carried off by the same kind of explosive inflammation, from very slight wounds in extremely hot weather. Practitioners who have seen such casas, declare that the whole system becomes immediately putrescent, on the invasion of the erysipelatous-looking inflammation and distension; that the blood becomes dissolved, and that the muscles do not stiffen before burial. Indeed, they say that the muscles become softened almost into a rotten pulp, and all the soft parts become detached from each other, and from the bones beneath in the affected limb. The only treatment which ever appeared to make any impression upon the constitution, consisted in a prompt evacuation of the stomach by ipecacuanha or mustard emetics, dashing buckets of cold water over the whole naked body, wrapping up the limb in sheets wrung out of cold water, and administering large doses of ammonia, either in the jorm of the carbonate or aromatic spirits. But even such a course has only been seen to give a temporary check to the progress of the tumefaction and sinking; the local disease has only been protracted thereby so as to go on to a more thorough degree of sphacelation. It would seem in this class of cases, as if the influence of injurious causes had not only rendered the solids excessively irritable and weak, but that they had also destroyed the vitality of the blood, or at least so far modified it as to induce a decided proneness to putrefaction."

The succeeding chapters treat of furunculus, anthrax, abscesses, ulcers, burns, effects of cold, &c. Then follow wounds, tetanus, hydrophobia, and poisoned wounds.

"So greatly do the effects of wounds received in dissection depend upon the present state of the system, that many sensible men believe there is no such thing as a true septic poison in dead bodies." At the opening of a course of dissections the student rarely suffers from a dissecting wound; but toward the close of the session the slightest puncture often produces alarming symptoms. Many of these cases evidently depend rather upon the condition of the system, than the character of the poison. Cases, however, do occur in which there can be no doubt of the reception of a peculiar and highly malignant poison; in regard to which two different forms are presented.

"By far the most dangerous and immediately fatal form, is that which results from the poison of subjects recently dead, and from whose blood the halitus or aura has not fully escaped. Some diseases are characterized by an odor as distinct from each other as occurs among different animals in health. The fomites of the clothing worn by patients who have labored under malignant fevers, is not more offensive or dangerous than the similar exhalation of a sickly and nauseous odor which so frequently offends us in recently exposed corpses. The post-mortem examination of all such cases should be postponed, not only until the offensive halitus has departed, but even until putrefaction has commenced. It is thonght by those who have investigated this matter, that putrefaction destroys the peculiar and malignant poison developed in the bodies of patients who die of certain diseases. The results of putrefaction itself, however, sufficiently often prove injurious to those who are exposed to them, and these constitute the second form of septic poison affecting the system through the medium of absorption. It is not necessary even that wounds should occur in order to render the human system liable to the injurious effects of putrefactive odors. They may be absorbed through the skin and sound mucous membranes, especially by the medium of respiration. All the washings and changes of clothing which dissecting room pupils may resort to, will never render them fit for entrance into general society. The effluvia which they necessarily imbibe during their filthy though praiseworthy occupations, will be exhaled for days afterwards from their persons either in the form of expiration or transpiration. Very debilitating effects, generally result in the constitutions of such individuals, and every precaution should always be taken by them to prevent the supervention of putrid fevers, or their associated forms of disease."

The treatment recommended by Dr. M'Lellan, is free suction applied to the wound, followed by nitrate of silver, sometimes preceding these measures by the application of a ligature.

"In the putrid diathesis resulting from absorption of morbid poisons, we may attempt to eliminate the putrescent matters by exciting all the secretions and exhalations, but it will rarely be proper to deplete in any shape. On the contrary, ammoniacal and violent stimuli will generally be required, along with opiates, and diluents, and nutritive broths. Great pains should always be taken to make timely and free incisions over all tumefied parts, and wherever we have reason to suspect the subjacent lodgment of matter."

We now come to that part of the subject of surgery upon which Dr. M'Clellan was always peculiarly interesting, viz. *Syphilitic poison.* In this chapter he criticizes freely the opinions of Ricord, while, at the same time, he awards him credit for some valuable contributions in this department. Hunter and Carmichael are, however, his favorite masters, the latter of whom claims that there are as many distinct varieties of virus as there are distinct forms of venereal disease; and that there are four varieties of virus, one producing the "papular venereal disease;" or the "lichen syphiliticus," the second producing the "pustular syphilis," the third the "tubercular," and the fourth the "scaly;" all of which are named from the symptoms presented in the secondary forms, and which, if traced back to their primary forms, will be found to have been preceded by distinctive primary symptoms. The first of these several varieties is "gonorrhoea!" which, contrary to the doctrine of Ricord and others, he declares to be capable occasionally of producing a genuine chancre—to be followed often by constitutional symptoms, resulting in the papular eruption, and, in short, to be a true syphilitic disease.

Dr. M'Clellan rejects, also, the classification adopted by Ricord, and prefers to speak only of primary and secondary symptoms. In the arrangement of these divisions he adopts the unusual mode of treating of the secondary symptoms first.

The "papular" form, or what we should call the *secondary class*, is to be managed with antiphlogistics mainly, and without mercury.

The "pustular" form, or the "phlyzaciom" of some writers, accompanied usually with white apthous ulcers in the fauces, is to be treated on nearly the same principles and without mercury, unless it be occasionally in the most chronic form.

The "tubercular" or "phagedenic," is a more formidable form than either of the preceding, and is accompanied with ulcers upon the skin, characterized by their having been preceded by a tubercle, healing from the centre while the circumference is extending, and in being covered often with thick conical scabs. The ulceration of the throat is also more rapid and alarming in its progress, destroying every structure in its course. With regard to the treatment, the author declares that he has never seen mercury cure or relieve these symptoms, but that, on the contrary, it never fails to exasperate them. Dr. Carmichael, and the British army surgeons, affirm that they have never seen nodes follow this form of syphilis except where mercury has been used, and Mr. Liston says, "this medicine may interrupt the progress of the disease, may remove the eruption and ulcers in the throat; but it, at the same time, transfers the disease to deep unyielding parts, to the bones and their coverings, and the fasciæ." The following excellent remarks we remember to have heard long since from Dr. M'Clellan himself.

"Since the publication of the earlier editions of Mr. Carmichael's work, a very great improvement has been made in the treatment of the primary symptoms of phagedena. Instead of the temporizing treatment recommended by him of blood-letting, fomentations, poultices, antimonials, purgatives and opiates, we now proceed immediately to destroy the whole morbid surface, by potential cauteries. A bridge of basilicon ointment is smeared around the edges, and over the surface of the neighboring skin, and then lint dipped in pure nitric acid is applied firmly over the sloughing or corroding surface. Emollient poultices afterwards speedily detach the sloughs and present a suppurating and granulating surface. In short, this efficient plan of treatment at once converts the diseased into a perfectly healty ulcer, and effectually protects the constitution from all contamination in most cases The secondary eruptions being accompanied with much febrile reaction, always require depletion at first, and generally, in the form of repeated blood-letting and purgatives. Antimonials and diluents afterwards come in as adjuvants, and finally, sarsaparilla with Dover's powder, or other sudorifics. The ulcers on the skin should be cauterized with caustic potass at their morbid edges and surfaces from time to time, and emollient poultices of slippery elm applied until the surface becomes healthy. Afterwards soap cerate, or Turner's cerate, spread on lint or linen, will speedily effect cicatrization."

Finally, the "scaly" form or the lepra syphilitica is that which Hunter regarded as the only genuine syphilis, the peculiar characteristics of which are sufficiently familiar to all; and it is here that mercury exhibits its greatest power, and that it becomes always proper and often indispensable.

Under this class the author arranges the "condylomata" of the English, or the "*tubercule muqueux*" of the French; the only form of secondary syphilis, which, since the time of Hunter, has been believed to be contagious. Ricord seems to think it may be contagious, while at the same time he has fully demonstrated that it is not "innoculable!" and Dr. M'Clellan assents to this doctrine. Mr. Liston, also, believes that a "secondary form of disease is occasionally communicated" from these tubercles; John Hunter demonstrated the same. Dr. M'Clellan, however, goes farther and thinks it by no means certain that other secondary sores than the mucous tubercle may not, under favorable circumstances, propagate the disease, since nearly all unhealthy secretions are found occasionally to propagate their kind. Perhaps after all, the profession will, sooner or later, come to the conclusion, that secondary syphilis is only not innoculable, or contagious (for we confess we have not sufficient metaphisical acumen to discover the distinction between these terms when applied to the propagation of syphilis) as a *general rule*, but that all of its forms under certain highly favorable conditions may be communicated by contact.

Exceptions are taken, not only to Ricord's classification of syphilis, but, also, to the general aphorism, the discovery of the truth of which is ascribed to the "French Hunter." It is not true that mercury is injurious in all the varieties of Ricord's tertiary syphylis; and that iodine is here a universal specific. Those forms of tertiary syphilis which assume the character of scrophula, doubtless demand iodine, but, even here, alterative doses of mercury will now and then more completely establish the cure. It is also certain that some tertiary ulcerations of the skin and mucous membrane are often benefitted by a judicious use of mercury.

We will not occupy more space by attempting an analysis of the primary symptoms and their treatment, yet this portion of the work is scarcely less interesting than the subjects just examined.

The book closes with the various forms of tumors, in the arrangement of which the author experiences the same difficulty which all who have attempted to teach, or write upon the subject, have experienced before him. Rejecting all precedents he becomes eclectic, and adopts so much of each as best suits his purpose. Under the head of non-malignant tumors, are arranged, encysted, cystoid and sarcomatous, of which latter there are the adipose sarcoma, or fatty, called by some, lipomatous, and of these the following varieties are named, the encysted, panniculated, the arborescent, the cholesteatoma and a peculiar congenital form described by Walther. The second class of sarcomatous tumors, viz, the fibro-cartilaginous tumors, or the enchondromata of Müller, constitute an order by themselves, as do also osseous tumors, under which last are arranged exostosis, spina ventosa, and simple osteo-sarcoma.

Under the head of malignant tumors are arranged first, malignant osteo-sarcoma, then the pseudo-plasmata, or malignant hetorologous tumors, presented in the following forms:—first, scrofula; second, scirrhus, scirrho-cancer or carcinoma; third, medullary cancer, medullary sarcoma, medullary fungus, encephaloid disease or soft cancer, fungoid tumor, fungus haematodes, most of which terms only express the varieties of one form. Finally melanosis, or black cancer completes the enumeration of malignant tumors; and after a full index comes "THE END;" if a book may be said to have an "end" which leaves off in the middle, and the other half of which was buried with its author. All that he might have said upon the great subjeet of operative surgery, and in which his practical tact, ingenuity, originality and skill always shone most conspicuously, is and must forever remain an almost complete loss. But sincerely as we regret for our own and humanity' sake this loss, we must be permitted to express our hope that no one will disturb the sacred repose of the revered M'Clellan by any vain attempts to resurrect and re-embody his thoughts. If it is indeed possible from the scattered fragments treasured in the memories and note books of his pupils, or from the thin, fugitive notices of his opinions and operations to be found in the journals, to again gather up and bring together the dry, marrowless bones of his giant conceptions, and so to make every

"joint embrace his kindred joint,"

yet who can be found to breathe upon this dead and senseless mass of matter and inspire it again with a living soul? The son of Dr. M'Clellan in giving to the public these manuscript pages of his honored father, has erected to his memory a lasting and appropriate monument. It was the sole remaining act of filial duty, and it has been promptly and religiously performed; but, as he values the reputation of his deceased parent, we conjure him to forego his plan of attempting to "carry out the work to the extent originally designed." We would wish to remember Dr. M'Clellan, and have the world know him hereafter, as his living instructions, and these pages written by his own hand, and glowing with the warmth and spirit of life, represent him. For *any* man to attempt a counterfeit of Dr. M'Clellan must inevitably prove a failure, and, our word for it, the world will never recognize or entertain the bastard claims of such a book.

